# Rhythmic potassium transport regulates the circadian clock in human red blood cells

**DOI:** 10.1038/s41467-017-02161-4

**Published:** 2017-12-07

**Authors:** Erin A. Henslee, Priya Crosby, Stephen J. Kitcatt, Jack S. W. Parry, Andrea Bernardini, Rula G. Abdallat, Gabriella Braun, Henry O. Fatoyinbo, Esther J. Harrison, Rachel S. Edgar, Kai F. Hoettges, Akhilesh B. Reddy, Rita I. Jabr, Malcolm von Schantz, John S. O’Neill, Fatima H. Labeed

**Affiliations:** 1 Department of Mechanical Engineering Sciences, Faculty of Engineering and Physical Sciences, University of Surrey, Guildford, Surrey GU2 7XH UK; 2 MRC Laboratory of Molecular Biology, Francis Crick Avenue, Cambridge, CB2 0QH UK; 3 Department of Biomedical Engineering, The Hashemite University, 330127, Zarqa, 13115 Jordan; 4 The Francis Crick Institute, 1 Midland Road, London, NW1 1AT UK; 5 Institute of Neurology, University College London, Queen Square, London, WC1N 3BG UK; 6 Faculty of Health and Medical Sciences, University of Surrey, Guildford, Surrey GU2 7XH UK

## Abstract

Circadian rhythms organize many aspects of cell biology and physiology to a daily temporal program that depends on clock gene expression cycles in most mammalian cell types. However, circadian rhythms are also observed in isolated mammalian red blood cells (RBCs), which lack nuclei, suggesting the existence of post-translational cellular clock mechanisms in these cells. Here we show using electrophysiological and pharmacological approaches that human RBCs display circadian regulation of membrane conductance and cytoplasmic conductivity that depends on the cycling of cytoplasmic K^+^ levels. Using pharmacological intervention and ion replacement, we show that inhibition of K^+^ transport abolishes RBC electrophysiological rhythms. Our results suggest that in the absence of conventional transcription cycles, RBCs maintain a circadian rhythm in membrane electrophysiology through dynamic regulation of K^+^ transport.

## Abstract (web summary)

Circadian rhythms usually rely on cyclic variations in gene expression. Red blood cells, however, display circadian rhythms while being devoid of nuclear DNA. Here, Henslee and colleagues show that circadian rhythms in isolated human red blood cells are dependent on rhythmic transport of K^+^ ions.

## Introduction

Circadian rhythms permeate biology, allowing organisms to anticipate the predictable daily cycle of night and day^1^. Our ~24 h sleep/wake cycle is an example of an overt output from this innate biological clock. In nucleated cells, this temporal program is realised as daily cycles of clock gene activity that drive rhythmic expression of clock-controlled genes in a tissue- and cell-type-dependent manner. Consequently, many aspects of cellular biology show circadian regulation, from metabolism and signal transduction to mitosis and differentiation. Clock disruption is strongly associated with chronic diseases, as well as deregulation of acute responses such as inflammation^2,3^.

Although coordinated between cells and tissues by humoral mechanisms, circadian rhythms are fundamentally cellular phenomena, with clock gene oscillations being readily observable in mammalian cells and tissues cultured ex vivo^4,5^. The prevailing model for timekeeping in mammalian cells comprises a transcription-translation feedback mechanism whereby cycles of transcriptional activation, expression, and auto-repression of clock genes such as *Period1* and *Period2*, and *Cryptochrome1* and *Cryptochrome2*, take ~24 h^6^. Recently, however, several observations have questioned whether transcription-translation feedback is absolutely required for circadian rhythmicity in mammalian and other eukaryotic cells^7–12^. In particular, circadian regulation of processes such as metabolism, redox balance, and proteasomal degradation persist in naturally anucleate, isolated human and mouse red blood cells (RBCs)^13,14^, suggesting that gene expression cycles are not essential for some form of molecular clockwork to persist. Thus far, a rhythm in the abundance of over-oxidised 2-Cys peroxiredoxin (PRX) proteins has served as the primary reporter for circadian timekeeping in RBCs. These cycles persist for at least 5 days ex vivo, are dependent upon the activity of the 20S proteasome^13^, and satisfy the technical criterion defining a circadian rhythm—a temperature compensated oscillation with a period of approximately 24 h.

On the basis of the catalytic cycle of PRX family members and their ancient evolutionary origins^15^, it has been suggested that these essential antioxidant proteins themselves constitute the basis of a self-sustained post-translational circadian redox oscillation^16^. Alternatively, and as observed in other contexts^17–20^, oscillations in PRX-SO_2/3_ abundance may be an indirect consequence, or epiphenomenon, of some as yet uncharacterised cellular timekeeping mechanism. In the latter case, one would expect to observe circadian regulation of other RBC activities that are not directly dependent on PRX activity.

Circadian regulation of transmembrane ion transport, whose initial discovery predates the identification of clock genes^21–23^, has recently become the subject of renewed interest in light of observations that cellular Mg^2+^ and K^+^ transport exhibit circadian rhythms in several different mammalian cell types, as well as in algae and fungal cells—suggestive of a circadian function with greater evolutionary conservation than any known clock gene^24,25^. In order to determine the post-translational timekeeping mechanisms at work within isolated RBCs, it is first necessary to understand whether circadian regulation extends to encompass any additional cellular systems beyond the previously described rhythms in PRX-SO_2/3_ abundance. Moreover, the way in which the RBC clock impacts upon physiologically relevant functions such as gas transportation, remains unexplored.

To this end, we tested the hypothesis that membrane ion transport processes might be circadian regulated in isolated human RBCs. Using dielectrophoresis and elemental analysis, we found evidence that K^+^ transport remains under clock control in the absence of underlying transcriptional cycles, or any external stimuli. Our findings establish dielectrophoretic assays as a tractable tool for delineating the RBC clock mechanism, as well as the consequences that circadian control of membrane electrophysiology might have in this most simple of all mammalian cell types.

## Results

### Electrophysiology reveals robust RBC circadian rhythms

Conventional methods for electrophysiology typically involve the use of patch-clamp to measure the dynamic electrical properties of cells. Patch-clamping is poorly suited to collecting robust measurements of electrophysiological changes in RBC populations over circadian timescales however, because of low processivity (due to small cell size) and the broad population distribution of resting membrane potential (~40% coefficient of variation). Instead, we have utilised dielectrophoresis, an alternative technique that permits rapid measurement of population-level electrophysiological properties in large numbers of cells simultaneously, and which moreover is readily compatible with circadian time course sampling in cell populations maintained under constant conditions over several days.

**Fig. 1:**
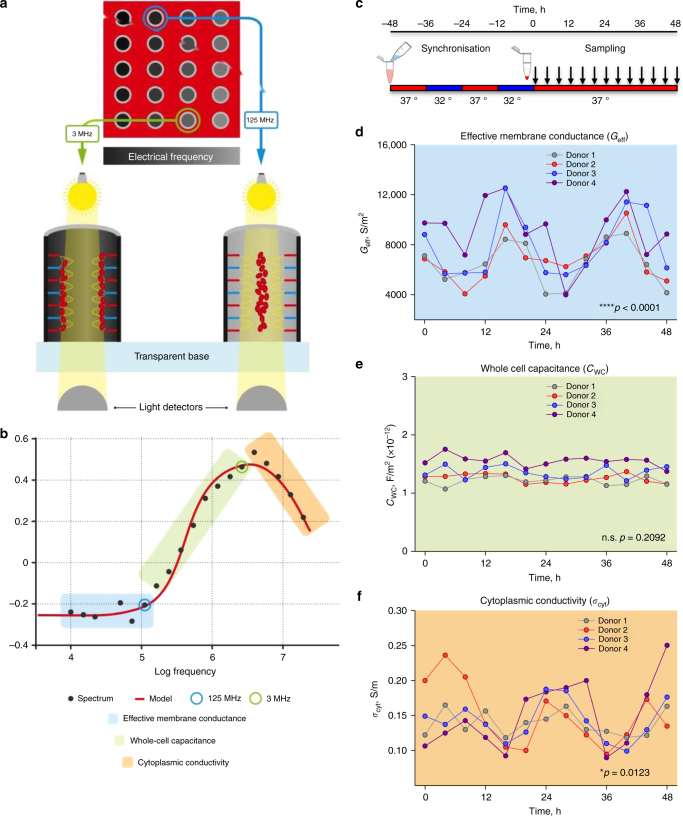
Circadian rhythms in human RBC electrophysiology are detected by dielectrophoresis. **a** Dielectrophoresis induces RBC movement towards the periphery (negative DEP) or centre (positive DEP) of each well as a function of applied AC frequency; this is measured by the variation in transmitted light intensity across each well. **b** Several electrophysiological parameters can be derived from this readout. **c** Human RBCs are isolated, and aliquots synchronised in parallel using 12 h:12 h 37 °C:32 °C temperature cycles over 2 days. RBCs are then maintained at constant 37 °C with a separate aliquot from each donor being analysed by DEP every 3 or 4 h over 2 days. **d** Effective membrane conductance (G_eff_) exhibits significant variation, *p* < 0.0001 for time effect by two-way ANOVA (*n* = 4, df = 12, *F* = 6.4). **e** Whole-cell capacitance (*C*_WC_) shows no significant variation over time, *p* = 0.2092 for time effect by two-way ANOVA (*n* = 4, df = 12, *F* = 1.4). **f** Cytoplasmic conductivity (*σ*_cyt_) also showed significant variation, *p* = 0.0123 for time effect by two-way ANOVA (*n* = 4, df = 12, *F* = 2.6)

Dielectrophoresis (DEP) is a technique in which non-uniform alternating electric fields induce cellular motion, dependent on both field frequency and cellular electrical properties^26^. The DEP system employed here measures the response of ~20,000 cells per test, using 20 different frequencies in parallel^27^ (Fig. 1a), and has been used in a range of cellular applications including drug discovery, stem cell characterisation, and the study of apoptosis^28,29^. Analysis of the frequency dependence of cell movement during DEP enables the determination of cellular electrophysiological parameters such as effective membrane conductance (*G*_eff_) per unit area, whole-cell capacitance (*C*_WC_), and cytoplasmic conductivity (*σ*_cyt_) (Fig. 1b). Whilst DEP has not previously been used for circadian assays, it has been employed to quantify changes in RBC electrophysiology in response to exogenous perturbations such as malarial infection^30^. On the basis of previous work in nucleated cells^23^, it seemed plausible that, in addition to circadian oscillations of metabolism and protein biochemistry^14^, ion transport (and thus membrane electrophysiology) might also be regulated by the intracellular clockwork in isolated RBCs.

Following the same synchronisation and sampling regime employed previously (Fig. 1c)^14^, DEP was used to measure *G*_eff_, *C*_WC_ and *σ*_cyt_ in RBCs isolated from four donors, and sampled every 3 h over 2 days under constant conditions. We observed significant, high-amplitude, *circa*-24 h changes in *G*_eff_, supporting the hypothesis that circadian regulation of ion transport might occur across the RBC membrane (Fig. 1d). *σ*_cyt_, indicative of ionic concentration inside the cytoplasm, also varied in antiphase with *G*_eff_. Although still statistically significant, the changes in *σ*_cyt_ were less consistent and of lower amplitude than *G*_eff_. By contrast, no significant variation was observed in a third, size/morphology-dependent parameter, *C*_WC_, confirming our observations by microscopy that cell radius (4.3 ± 0.4 µm) and morphology did not change over the course of RBC incubations (Fig. 1e).

**Fig. 2:**
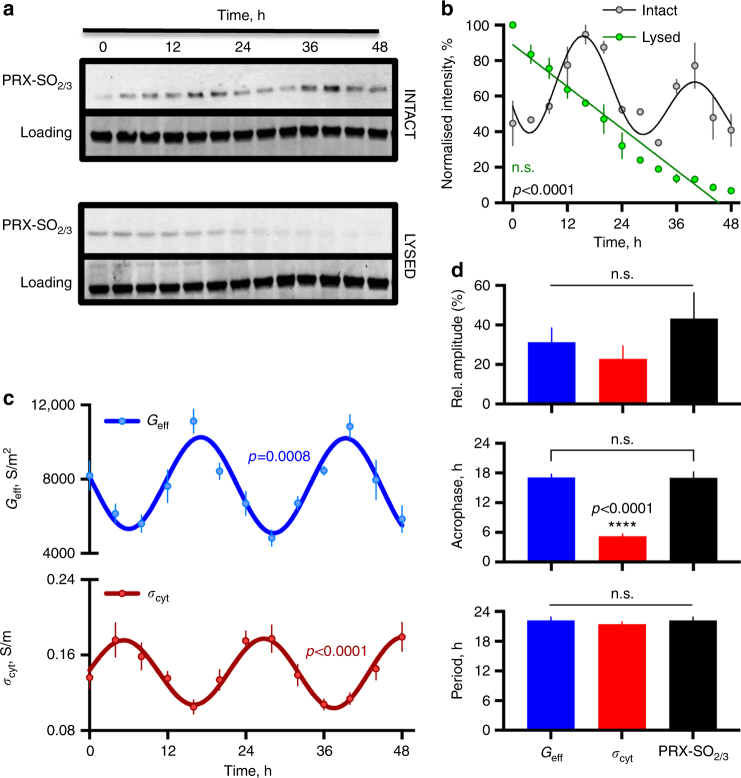
Effective membrane conductance and over-oxidised peroxiredoxin levels peak during the anticipated cold phase. **a** In human RBCs, peroxiredoxin over-oxidation abundance rhythms peak during the anticipated cold phase following temperature cycles (as in Fig. 1c) and are abolished by RBC lysis. Representative blots are shown (*n* = 3). **b** Western blot quantification of PRX-SO_2/3_ levels in intact vs. lysed human RBCs (mean ± SEM, *n* = 3), p < 0.0001 for interaction effect by 2-way ANOVA (*n* = 3, df = 12, *F* = 12.2). **c** Effective membrane conductance also peaks during the anticipated cold phase, in antiphase with cytoplasmic conductivity; mean ± SEM, *n* = 4. **d** No significant difference between the periods and relative amplitudes were detected between the two rhythmic DEP parameters and PRX-SO_2/3_ (mean ± SEM, *n* = 4 and 3, respectively *p* = 0.59 by one-way ANOVA, df = 11, *F* = 0.55), whereas a significant difference in phase was detected (Holm–Sidak post-test). Rhythmicity and parameters were determined by cosinor vs. straight line fit (*R* ^2^ > 0.6, *p* value of fit comparison reported in each case for **b** and **c**)

We next validated and compared the PRX-SO_2/3_ oscillations in isolated human RBCs with electrophysiological rhythms measured by DEP under identical conditions. We observed peak PRX-SO_2/3_ to be roughly coincident with peak *G*_eff_, during what would be the anticipated cold phase based on prior temperature cycles (Fig. 2a–c). We observed no significant oscillation in lysed RBC controls (Fig. 2a, b). Importantly, no difference in circadian period or relative amplitude was detected between *G*_eff,_ *σ*_cyt_ and PRX-SO_2/3_ (Fig. 2d). Furthermore, *G*_eff_ and *σ*_cyt_ again oscillated in antiphase with each other (Fig. 2d), supporting the parsimonious interpretation that these two electrophysiological parameters represent opposing facets of the same ion transport process. In other words, the steady state ionic content of the cytoplasm, which determines cytoplasmic conductivity (*σ*_cyt_), is lowered by an increased net rate of ion efflux that occurs in isolated RBCs during the subjective night with respect to the entraining temperature cycle (higher body temperatures normally occur during the day in humans).

### K^+^ transport underlies RBC electrophysiological rhythms

**Fig. 3:**
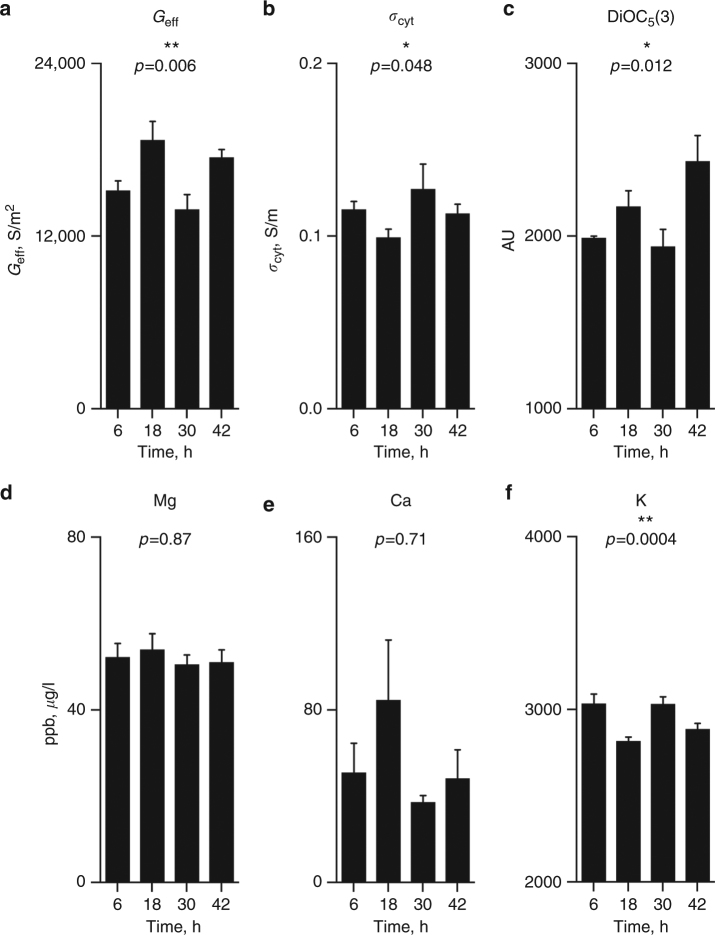
Validation of circadian DEP measurements using a membrane potential sensitive dye and ICP-MS. Following synchronisation, after 18 h at constant 37 °C, G_eff_ is significantly increased (**a**), and *σ*_cyt_ significantly decreased (**b**), compared with 12 h before or afterwards, as shown in Figs 1 and 2. In RBCs sampled at these time points, we observed a significant increase in membrane potential (**c**), reported by a voltage-sensitive membrane dye (DiOC_5_(3)), and a significant decrease in cellular K content (**f**), as determined by ICP-MS. Other biologically relevant elements did not change significantly (see examples **d** and **e**, as well Supplementary Fig. 1). Note that intracellular Na^+^ and Cl^−^ could not be determined accurately by this method due to their high extracellular concentrations. Mean ± SEM reported throughout, *n* = 4 biological replicates, *p* values indicate Kruskal–Wallis significance test

In light of the antiphasic rhythm of *G*_eff_ compared with *σ*_cyt_, we thought it likely that differential regulation of ion transport might be responsible for the observed DEP rhythms. To test this, alongside DEP, we performed inductively coupled plasma-mass spectrometry (ICP-MS), as well as flow cytometry with a voltage-sensitive dye, at time points coincident with the *G*_eff_ minima and maxima (Fig. 3a, b). Membrane potential, as measured by the membrane dye DiOC_5_(3)^17^, was significantly greater at 18 h than was observed 12 h before or afterwards (Fig. 3c), corresponding with the time of peak *G*_eff_ and nadir of *σ*_cyt_. By ICP-MS analysis of identical samples in parallel at the same time points, we observed a significant variation in total cellular K^+^ content (Fig. 3d) that was lowest at 18 h and coincident with maximal *G*_eff_ and membrane hyperpolarisation. Importantly, we observed no significant variation in the cellular concentration of other biologically relevant ions (Fig. 3e, f, Supplementary Fig. 1), although it is important to note that for technical reasons intracellular Na^+^ and Cl^−^ could not be measured as accurately as other ions due to their much higher extracellular concentration.

**Fig. 4:**
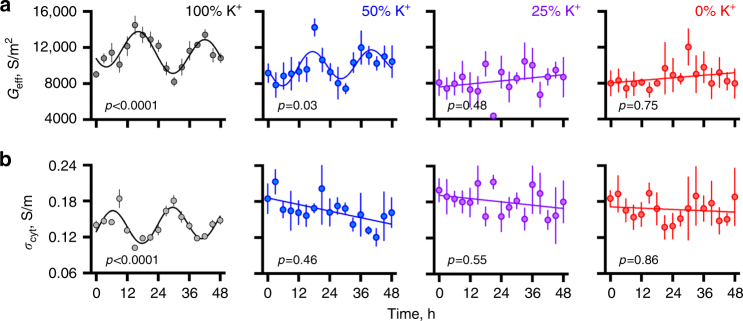
Circadian electrophysiological rhythms in isolated human RBCs depend upon extracellular [K^+^]. Circadian rhythms of *G*_eff_ (**a**), and *σ*_cyt_ (**b**), in isolated RBCs were not detected when the concentration of extracellular K^+^ was ≤25% of its normal concentration (5 mM). Mean ± SEM reported, *n* = 4 biological replicates, *p* values report the probability that data series are better described by a damped cosine model in preference to the null hypothesis (straight line); for *p* > 0.05 the straight line fit is shown, for *p* < 0.05 the damped cosine fit is shown (solid line)

The electrochemical potential gradient of K^+^ over the plasma membrane is one of the principal determinants of resting membrane potential in mammalian cells, and we thus deemed it likely that rhythmic regulation of K^+^ transport underlies the rhythm in RBC electrophysiology observed using DEP and FACS. To test this, we repeated the DEP time course experiments in medium that was depleted for K^+^ (usually 5 mM). We observed a K^+^-dependent loss of amplitude for the rhythms in both *G*_eff_ and *σ*_cyt_ (Fig. 4), with no significant circadian rhythm being observed in either parameter when extracellular [K^+^] was ≤25% of its normal extracellular concentration. Importantly, RBCs maintained viability and cell morphology over 4 days in K^+^-depleted media, and our findings therefore support a model in which circadian RBC membrane rhythms are dependent upon differential K^+^ transport.

We next investigated the relationship between electrophysiological rhythms in RBCs, detected by DEP, with those in rhythms of PRX-SO_2/3_ abundance. PRX-SO_2/3_ rhythms are abolished by proteasomal inhibition using MG132^13^; we found the same to be true for DEP rhythms, whereas selective inhibition of PRX activity (with conoidin A)^17^ attenuated DEP rhythms, but did not eliminate them. Reciprocally, we found that RBC circadian PRX-SO_2/3_ abundance changes were attenuated but not immediately abolished in K^+^ depleted medium—when DEP rhythms were not observed (Supplementary Figs 2 and 4). Therefore, ion transport and PRX-SO_2/3_ rhythms are affected by, but not directly dependent on, each other, and may therefore be linked indirectly. Similar to recent findings in cultured mouse and human cell lines^19,24^, these results suggest that neither PRX and ion transport activity are true state variables of the circadian clock in RBCs, but interact with a cellular timekeeping system whose outputs and/or mechanism is sensitive to proteasomal inhibition.

### K^+^-transport affects the RBC timekeeping mechanism

Circadian regulation of K^+^ transport has been observed across a wide range of nucleated eukaryotic cell types and is thought to be the major factor driving electrical excitability rhythms in the mammalian master clock—the hypothalamic suprachiasmatic nuclei (SCN). In both SCN and *Drosophila* pacemaker neurons, as well as non-excitable fibroblast cells, plasma membrane potential feeds back to modulate the cellular clock mechanism itself^31–34^. To test whether K^+^ transport rhythms make any functional contribution to the RBC clock that we observed using DEP, we performed pharmacological manipulations that increased or inhibited K^+^ efflux in the expectation that these treatments would have differential effects upon DEP rhythms and their baseline values.

**Fig. 5:**
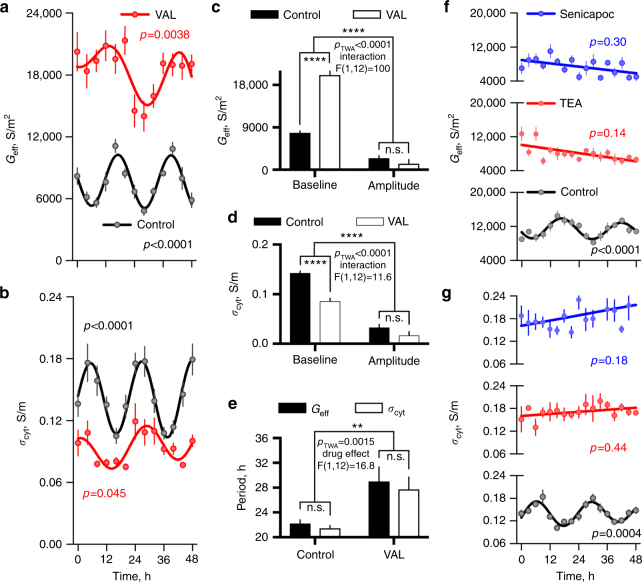
RBC circadian rhythms are affected by perturbation of K^+^ transport. The data points report DEP measurements of *G*_eff_ (**a**), and *σ*_cyt_ (**b**), in isolated RBCs over 2 days in the presence of 30 nM valinomycin or vehicle control (mean ± SEM, *n* = 4 biological replicates, cosine fit *p* value reported). **c**–**e** Valinomycin treatment had significant effects on baseline *G*_eff_ and *σ*_cyt_ as well as lengthening circadian period of oscillation measured for both DEP parameters, without affecting the amplitude of their oscillation; two-way ANOVA *p* values (*p*_TWA_) and *F* statistics for interaction or drug effect are indicated, as well as selected Fisher’s post-test *p* values. **f**, **g** The data points report DEP measurements of *G*_eff_ and *σ*_cyt_ in isolated RBCs over 2 days in the presence of 11 nM senicapoc, 15 mM TEA or vehicle control, mean ± SEM, *n* = 4 biological replicates. *P* values in graphs **a**, **b**, **f** and **g** indicate report the probability that data series are better described by a damped cosine model than the null hypothesis (straight line); for *p* > 0.05 the straight line fit is shown, for *p* < 0.05 the damped cosine fit is shown (solid line)

We first repeated the DEP time course assays in the presence of the K^+^ ionophore valinomycin (VAL). Intracellular [K^+^] in erythrocytes is around 140 mM, much higher than found extracellularly (5 mM), and we predicted that valinomycin-mediated increases in K^+^ efflux would increase *G*_eff_ as measured by DEP, with a concomitant reduction in *σ*_cyt_. For this experiment, we employed the highest concentration of valinomycin (30 nM) that did not result in detectable changes in RBC morphology or viability over 4 days, in order that we might also assess whether increased K^+^ efflux had any effect on RBC circadian rhythms compared with controls. In the presence of valinomycin, we observed that baseline *G*_eff_ was increased approximately two-fold, along with reduced mean cytoplasmic conductivity (Fig. 5a–d), indicating that differential K^+^ transport is likely to contribute to circadian regulation of RBC electrophysiology as we expected. We also observed that whilst a significant rhythm was detected for both *G*_eff_ and *σ*_cyt_ in the presence of valinomycin, the apparent period of oscillation was lengthened by 6 ± 2 h for both DEP parameters. To our knowledge, this constitutes the first example of an exogenous manipulation that affects the period of the RBC clock (Fig. 5a, b, e).

We next performed the converse manipulation: pharmacological blockade of K^+^-efflux. For this, we used tetraethylammonium (TEA), a broad-spectrum competitive inhibitor of K^+^ channels, or senicapoc, a selective and potent inhibitor of Gardos channel activity^35^, which acts by blocking the primary means of K^+^ efflux over the RBC membrane. Again we employed the highest concentrations that did not affect cell viability or cell morphology over 4 days. Chronic incubation with either drug phenocopied the effect of K^+^ depletion upon RBC electrophysiological rhythms, i.e., no significant circadian oscillation was observed (Fig. 5f, g). Both treatments were also associated with increased basal *σ*_cyt_, which is consistent with increased accumulation of intracellular K^+^ due to efflux blockade.

Taken together, the data presented in Figs 3–5 indicate that differential regulation of K^+^-transport underlies the circadian rhythms of RBC membrane physiology rhythms we detected using DEP, and also suggest perhaps K^+^ transport may contribute to determination of circadian period in isolated RBCs.

## Discussion

We observed RBC electrophysiological rhythms by DEP, validated them using a voltage-sensitive fluorescent membrane dye and then, by ICP-MS, observed that cellular K^+^ levels varied in synchrony with DEP parameters. Cell-autonomous circadian rhythms in cellular K^+^ content were recently described in cultured mammalian cell lines as well as algal and fungal circadian cellular models^18^, suggestive of an evolutionarily conserved clock mechanism^17^. Since ion channel activity can be regulated post-translationally, we pursued the hypothesis that differential regulation of K^+^ transport is contributes to circadian regulation of RBC electrophysiology. In support of this, we found that both depletion of extracellular K^+^ as well as the use of K^+^ channel blockers (TEA and senicapoc) abolished RBC rhythms in the DEP parameters *G*_eff_ and *σ*_cyt._ Interestingly, VAL treatment did not abolish the RBC DEP rhythm but substantially increased *G*_eff_ and reduced *σ*_cyt_. This is consistent with dynamic regulation of both transmembrane K^+^ efflux (TEA) and influx (K^+^ depletion) being critical to rhythmic RBC membrane electrophysiology. Rhythmic transmembrane transport of K^+^ ions also occurs in the marine picoeukaryote, *Ostreococcus tauri*, and persists in the absence of transcription (under constant darkness)^24^. Thus, circadian control of membrane electrophysiology in eukaryotes can be regulated post-translationally and occur without requiring circadian cycles of transcriptional activation and repression.

Clock control of Na^+^/K^+^ transport is viewed as being essential to membrane excitability rhythms in the central pacemakers of flies and mice, and feedback to regulate core clock gene expression cycles, making membrane excitability essentially indistinguishable from a core clock component. RBCs are non-excitable cells, and do not express cycles of clock gene transcription since they lack a nucleus. While the clock mechanism in RBCs remains unclear, it seemed plausible to us that rhythmic regulation of transmembrane ion transport in RBCs might similarly contribute to, or at least influence, whatever mechanism(s) determine the period of oscillation. In support of this, we found low concentrations of the K^+^ionophore valinomycin, which acts to increase the rate of K^+^efflux, lengthened the apparent circadian rhythm in both DEP parameters-while their antiphasic relationship was maintained. This supports the possibility that transmembrane ion transport may facilitate or contribute towards the RBC clock mechanism itself. Future work will need to determine the specific ion channels responsible for circadian K^+^ transport in RBCs, which post-translational processes impart the observed circadian conductance rhythm, and whether rhythmic K^+^ transport also contributes to timekeeping in other non-excitable mammalian cell types^18^.

On the basis of previous observations with RBCs, coupled with older indications in the literature^18^, we investigated DEP as an alternative platform for probing the transcription-independent cellular clock in isolated human RBCs. DEP allows measurement on short timescales and with limited preparation time, enabling the detection of robust and quantitative circadian rhythms in membrane electrophysiology and cytoplasmic conductivity to be observed. Although DEP does not measure resting membrane potential directly, measurement of cytoplasmic conductivity (largely indicative of conduction by cations such as K^+^, Na^+^ and Ca^2+^) does allow relative changes in membrane potential to be inferred^36^. A coupled circadian oscillation was observed between *G*_eff_ and *σ*_cyt_. There was no significant change in a third parameter, *C*_WC_, indicating that there was no change in membrane morphology or cell size, and hence eliminating this as a potential variable underlying our observations. While we do not discount the possibility that subtle changes in cell morphology or volume could occur, we have not detected them by quantitative microscopy, and more importantly, they would not be able to account for the effects that we observe. Incidentally, this also suggests that *C*_WC_ is a useful internal negative control in DEP assays of RBC circadian rhythms.

Because RBCs constitute the most abundant cell type in the human body, and have a vital function, it will be important to establish the physiological significance of circadian regulation of RBC ion transport, and whether its disruption has any pathophysiological consequence. For example, RBCs play an essential role in transporting CO_2_ from tissues to the lungs, which in turn is dependent upon the activity of the Band 3 bicarbonate transporter (SLC4A1) whose activity is sensitive to membrane potential^37^. It should now be tested whether circadian regulation of RBC membrane physiology affects RBC CO_2_-carrying potential. Likewise, circadian rhythms in RBCs may be an unexplored factor contributing to the strong diurnal pattern to the onset of myocardial infarction and other adverse events of the cardiovascular system^38^. Moreover, *Plasmodium* infection of RBCs in malaria is accompanied by marked changes in cell electrophysiology^30^, and it is therefore possible that RBC electrophysiological rhythms contribute to the diurnal variability in cycles of malarial infection and replication.

A further question concerns what adaptive advantage might be conferred upon RBC function by being able to anticipate the circadian organisation of organismal physiology. We speculate that cell autonomous circadian regulation of ion transport may help to counteract the increased osmotic burden to which blood cells are subject at night, when serum protein levels fall in vivo^39^. RBCs cannot accommodate this decrease in water potential by changes in the expression of transporters in the plasma membrane (as can other cells of the haemopoetic lineage), and yet cytosolic osmolality must be defended in order to prevent protein aggregation due to molecular crowding^40^. Therefore, we suggest that increased K^+^ efflux at night would act to reduce the hyperosmotic stress to which the red cell interior would otherwise be subject. It remains to be seen whether cation transport rhythms observed in other (nucleated) cell types^41,42^ also might contribute to osmotic homeostasis.

**Fig. 6:**
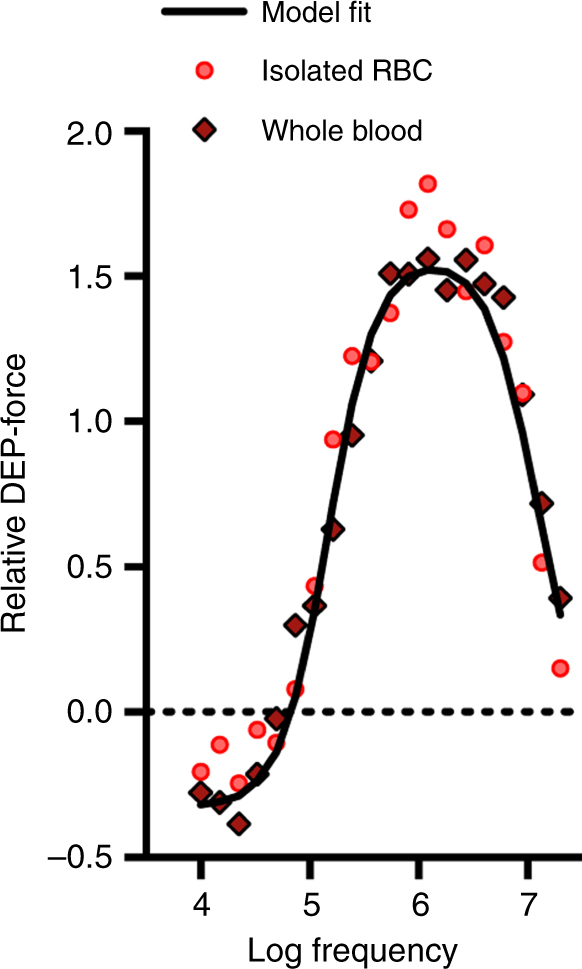
Dielectrophoretic properties of acutely sampled whole human blood. Comparison of DEP spectra produced from isolated RBCs compared with whole blood from the same donor (representative spectra from a single donor are shown). Isolated RBCs were prepared as described and tested immediately following isolation. DEP model fit parameters showed no significant variation (*p* > 0.05 RM one-way ANOVA for sample type effect, df = 2, *F* = 3.51) between the isolated and whole blood samples, meaning that DEP may have diagnostic utility as an acute diagnostic marker for human circadian phase

Finally, we note that unlike melatonin, which is detected by radioimmunoassay, DEP assays are performed in minutes. Consequently, DEP may have potential as a circadian phase marker^43^. RBCs make up around 99% of circulating blood cells, and thus it is possible that accurate measurements could be made from acutely sampled whole blood, without requiring purification to remove white blood cells. Certainly, the model used to fit DEP spectra of isolated RBCs cannot distinguish between isolated RBCs and whole blood sampled acutely from the same donor (Fig. 6). If the circadian electrophysiological rhythms in isolated RBCs also occur in vivo, DEP may be of diagnostic utility. In future, it will be important to determine whether RBC electrophysiology shows diurnal variation in blood from healthy individuals under natural lighting conditions over several days, as well as under constant conditions.

## Methods

### Human participants

Studies were conducted in accordance with the principles of the Declaration of Helsinki, with approval/favourable opinion from the Local Research Ethics Committee (Universities of Cambridge and Surrey, UK). Participants in the study were screened for relevant self-reported health issues, including sleep disorders or excessive daytime sleepiness. All participants provided written, informed consent after having received a detailed explanation of the study procedures.

### Human RBC isolation

RBCs were isolated from anticoagulant-treated whole blood by density gradient centrifugation layering a mixture of whole blood and PBS (1:3) on top of Histopaque-1077 (1.077 g/ml polysucrose solution) (Sigma-Aldrich, St Louis, MO) according to manufacturer’s instructions. RBCs were washed twice in PBS before resuspension in Krebs–Henseleit buffer (KHB, pH 7.4, 290 mOsm) (Sigma-Aldrich)^14^. These minimal medium conditions ensure the tiny fraction of nucleated cells detectable in RBC pellets immediately after centrifugation (~0.02%) undergo cell death in <24 h and cannot influence RBC circadian rhythms^14^. To corroborate this, we tested whether an additional granulocyte-depletion step with anti-CD15 Dynabeads (Life Technologies, Carlsbad, CA), with depletion confirmed by gel zymography, had any effect upon RBC rhythms. We observed no difference between those RBCs and RBCs isolated without CD15 depletion (Supplementary Fig. 3). Circadian entrainment was achieved by 12:12 h 32 °C:37 °C temperature cycles (mimicking the endogenous cycle of body temperature rhythms that helps to synchronise cells throughout the body in vivo) over 2 days using a thermal cycler, and then incubated them at constant 37 °C for time course sampling, with a separate aliquot being removed from the cycler for analysis at each time point for each donor, i.e. following the same procedure as employed in ref. ^14^. In these experiments, the final transition to 37 °C is taken as *t* = 0 (or Zeitgeber time 0, ZT0). In all experimental time courses, medium osmolality was measured and adjusted to 290 mOsm. For haemolysis, RBC pellets were lysed using freeze-thaw cycles, or by resuspension in an equal volume of distilled water, or by resuspension in an equal volume of KHB + 0.1% SDS, and were then diluted to the same volume as intact cell controls, with normal or slightly concentrated KHB, to give 1× Krebs buffer. In each case, haemolysis was clearly evident. The data from SDS lysis is presented in Fig. 2, being representative of the other lysis conditions.

### Gel electrophoresis and immunoblotting

At each time point, the red cell pellet was resuspended by trituration and 50 μl was removed and added to 450 μL 2× LDS sample buffer (Life Technologies) supplemented with 5 mM DTPA and snap frozen in liquid N_2_, then stored at −80 °C. At a later time, samples were thawed on ice then heated at 70 °C for 10 min on a shaking heat block. Western blotting and SDS-PAGE were performed using the NuPage Novex midi system (Life Technologies) with 4–12% bis-tris gradient gels and nitrocellulose transfer stacks according to the manufacturer’s instructions. Blots were blocked in 0.5% (w/w) BSA/non-fat dried milk (Marvel) dissolved in Tris-buffered saline/0.05% Tween-20 for 1 h at room temperature, and then incubated overnight at 4 °C on a rotary shaker with 1:10000 ab16830 diluted in blocking buffer. Blots were washed for 3 × 5 min in TBST then incubated for 1 h with a 1:10000 dilution of HRP-conjugated anti-rabbit secondary, then washed for 4 × 10 min, with TBST before chemiluminescence detection was performed using Immobilon (Millipore, Billerica, MA) according to manufacturer’s instructions^44^. Full uncropped scans of western blots are presented in Supplementary Fig. 4.

### Dielectrophoresis

After the 48 h entrainment, RBC pellets were removed at 4 h intervals for the assessment of cell electrophysiology by dielectrophoresis (DEP). The Krebs buffer was removed and the pellet was suspended in iso-osmotic DEP medium^45^ adjusted to a conductivity of 0.043 s/m using phosphate-buffered saline (PBS, Labtech International, Heathfield, UK), and resuspended at a final concentration of 10^6^ cells per ml (Fig. 1c). Cell suspensions were pipetted into 3DEP chips (DEPtech, Heathfield, UK), which were subsequently inserted into a 3DEP reader (DEPtech), where pin connections energised each well at 10 V_p–p_, with a different frequency applied to each one of the 20 wells, and the wells were collectively energised for 10 s at five points per decade (10 kHz—20 MHz) (Fig. 1a). This was repeated at each time point for each donor at least four times. The raw data were fitted with a single-shell model in order to extract the electrophysiological parameters^27,46^, accepting spectra producing *R* ^2^ values of 0.9 or greater. For whole blood DEP spectra (Fig. 6), finger pricks were conducted with sterile lancets and 10 µl whole blood was collected in coated capillary tubes and immediately transferred to 5 ml modified DEP medium, adjusted to control for the slight increase in conductivity due to blood plasma that would otherwise result. For dielectrophoresis analysis, data set modelling producing an *R* value (Pearson correlation coefficient) of 0.9 or less were excluded.

### Pharmacological treatments

For each drug, cell viability and morphology were initially screened over a period of 4 days using a range of concentrations. In each case, the concentration used for subsequent time course experiments, adapted from Bratosin et al.^47^, was the highest, which did not result in haemolysis, altered viability or morphology under our experimental conditions (see Supplementary Fig. 5 for example). The drugs used in this study included, 30 nM valinomycin (Sigma-Aldrich) 15 mM tetraethylammonium chloride-TEA (Sigma-Aldrich), 11 nM senicapcoc (Insight Biotechnology, Wembley, London, UK). In each case, an equivalent % of DMSO vehicle was used as a control. Drug addition to RBC suspensions was performed immediately after the 48 h entrainment that preceded each 48 h time course (under constant conditions), where DEP analysis began at ZT0, with sampling from a fresh aliquot of RBCs every 3 or 4 h.

### Ion Exclusion

For each ion concentration (0, 25 and 50%), cell viability and morphology were initially screened over a period of 4 days using a range of concentrations. In each case, the concentrations used for subsequent time course experiments were those that did not result in haemolysis, altered viability or morphology under our experimental conditions. Percentage concentrations were determined from the amount of ions compared to the control KHB buffer. For K^+^ depletion, the 5 mM of KCl in control buffer was replaced with 5 mM of NaCl. Percentages were then established from mixtures of control and exclusion media. RBCs were isolated and prepared as described in the substitute media and sampled every 3 h post entrainment.

### FACS Analysis

RBCs were isolated and entrained as described above in quadruplicate for DEP, ICP-MS (x2) and FACS analysis. Stock solution of DiOC_3_(5) (Stratech Scientific, Newmarket, Suffolk, UK) was made to 1 mM in DMSO with a working stock solution in PBS at 1.5 µM. Pellets were resuspended in the working stock at a concentration of 10^6^ cells per ml and left to incubate for 25 min. RBCs were then washed twice with PBS for 5 min at 1300 r.p.m. Fluorescence measurements were carried out on an Attune Flow Cytometer (Thermo Fisher, Waltham, MA). Cells were gated against volume vs. scatter.

### ICP-MS

RBCs were prepared in duplicate for ICP-MS analysis. Upon removal from PCR, tubes were placed on ice. Cells were washed in ice-cold iso-osmotic media containing: 8.5% w/v sucrose, 0.3% w/v dextrose (both from Sigma-Aldrich), 5 mM HEPES at pH 7.4. Suspensions were centrifuged at 3 °C at 3000 r.p.m for 3 min and placed on ice. Exactly 480 µl of buffer was removed from the formed pellets and immediately placed in −80 °C freezer. RBC pellets were then lysed in 550 μL 65% HNO_3_ supplemented with 100 p.p.b. Ce overnight at room temperature then diluted 1:12 in HPLC-grade water to give a final matrix concentration of 5% HNO_3_. ICP-MS was performed on a Elan DRC II (Perkin Elmer, Waltham, MA), with SPS-SW2 (LGC) being used as a routine standard^24^. Cerium in the HNO_3_ extraction reagent was used to correct for dilution errors introduced during handling.

### Statistical Analysis

Time course data were determined to remove baseline changes and fit with a damped cosine in order to determine the circadian parameters period, amplitude and phase as described by Hirota et al.^48^. All graphs and analyses were performed in Prism 6 (Graphpad Software, La Jolla, CA). Analyses are reported in figure legends. To determine whether a circadian rhythm was present, a straight line fit (*y* = m*x* + *c*) was compared with a damped cosine + baseline fit (*y* = *mx* + *c* + amplitude×e^(-*kx*)^×cos(2*π*×(X-phase)/period)) using the extra sum-of-squares F test in the compare models function of Prism 6, with the simpler model being preferred unless the *p* value was <0.05. Relative amplitude is amplitude/baseline. All tests are two-sided and “*n*” always refers to the number of biological replicates.

### Data availability

All the relevant data can be available on request from the corresponding authors.

## Electronic supplementary material

Supplementary Information
