# Supplementary material for: Rhythmic potassium transport regulates the circadian clock in human red blood cells
Source: Nat Commun. 2017 Dec 7;8:1978. doi: 10.1038/s41467-017-02161-4 (PMC5719349; doi:10.1038/s41467-017-02161-4)
Supplement: Supplementary file 1 — Supplementary Information [file 41467_2017_2161_MOESM1_ESM.pdf]

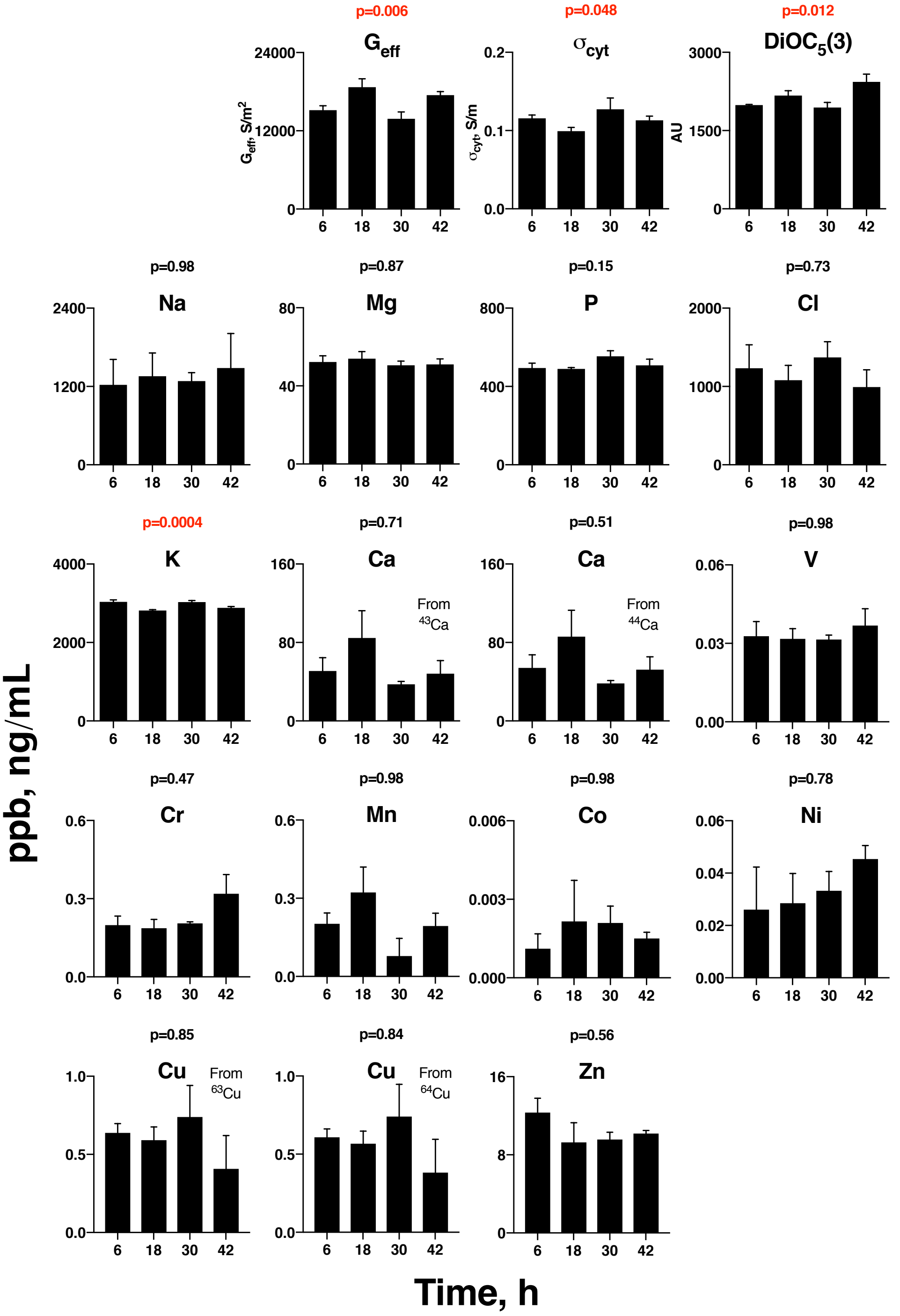

Supplementary Figure 1. Validation of DEP measurements using a membrane dye and ICP-MS (expanded from Figure 3)

Following synchronisation, after 18 h at constant 37 degrees, G_{eff} is significantly increased and σ_{cyt} significantly decreased compared with 12 h before or afterwards, as shown in Figures 1 & 2. In RBCs sampled at these time points we observed a significant increase in membrane potential reported by a voltage-sensitive membrane dye, and a significant decrease in cellular K content as determined by ICP-MS. Other biologically relevant elements did not change significantly using this assay; p-values indicate Kruskal-Wallis significance test, n=4 biological replicates. Please note however, that due to their high extracellular concentrations there was increased error associated with the measurement of intracellular Na^+ and Cl^-, compared with other analytes. More sensitive methods will need to be developed in order to determine whether intracellular Na^+ and/or Cl^- concentration is also subject to circadian regulation.

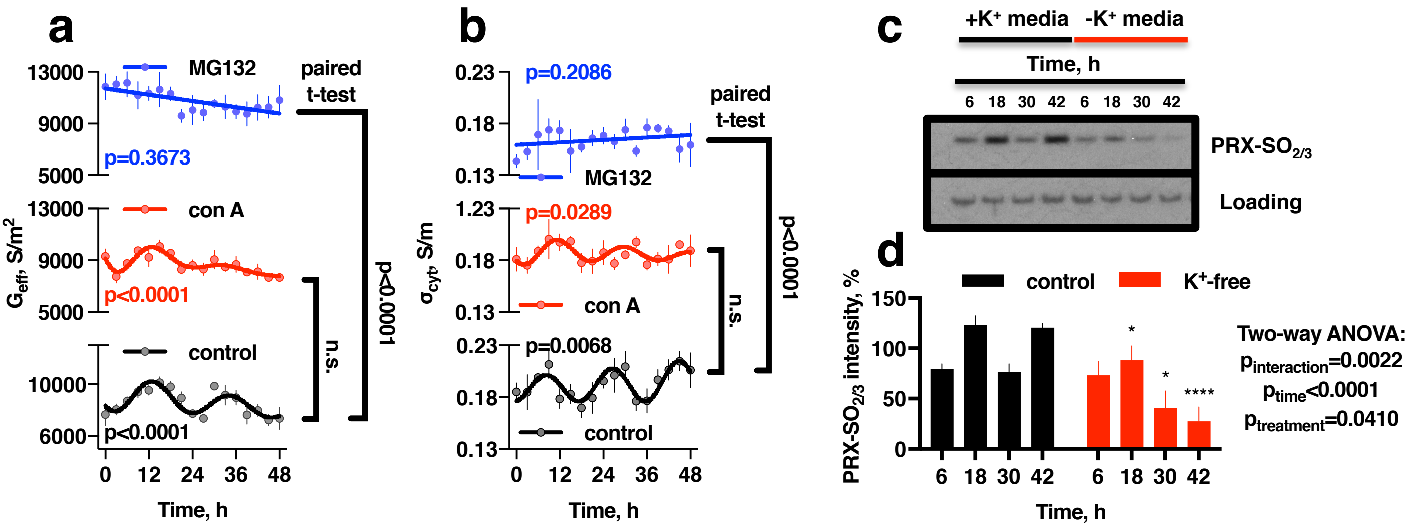

Supplementary Figure 2. Indirect relationship between circadian DEP and $PRX\text{-}SO_{2/3}$ abundance rhythms in isolated human RBCs.

Circadian rhythm of G_{eff} (A) and σ_{cyt} (B) persists during PRX inhibition with 5 µM conoidin A, but is abolished by proteasomal inhibition with 3 µM MG132, a treatment that also abolishes $PRX\text{-}SO_{2/3}$ rhythms in isolated RBCs[13]; data points are mean ± SEM (n=4). Solid lines show cosinor or straight line fit to each time series with the cosinor fit only preferred if $p<0.05$, specific p-value of fit is reported on the left hand side; paired t-test values comparing the data points at each time point between drug treatment and vehicle controls are reported on the right hand side. (C) Representative western blot and quantification (D) showing attenuation of $PRX\text{-}SO_{2/3}$ abundance cycles in K^+-depleted media, which also abolishes DEP rhythms (Figure 4); mean ± SEM (n=4) with two-way ANOVA p-values reported as well as Fisher's post-test p-values for K^+-free vs. control media at each time point (*$p<0.05$, ****$p<0.0001$).

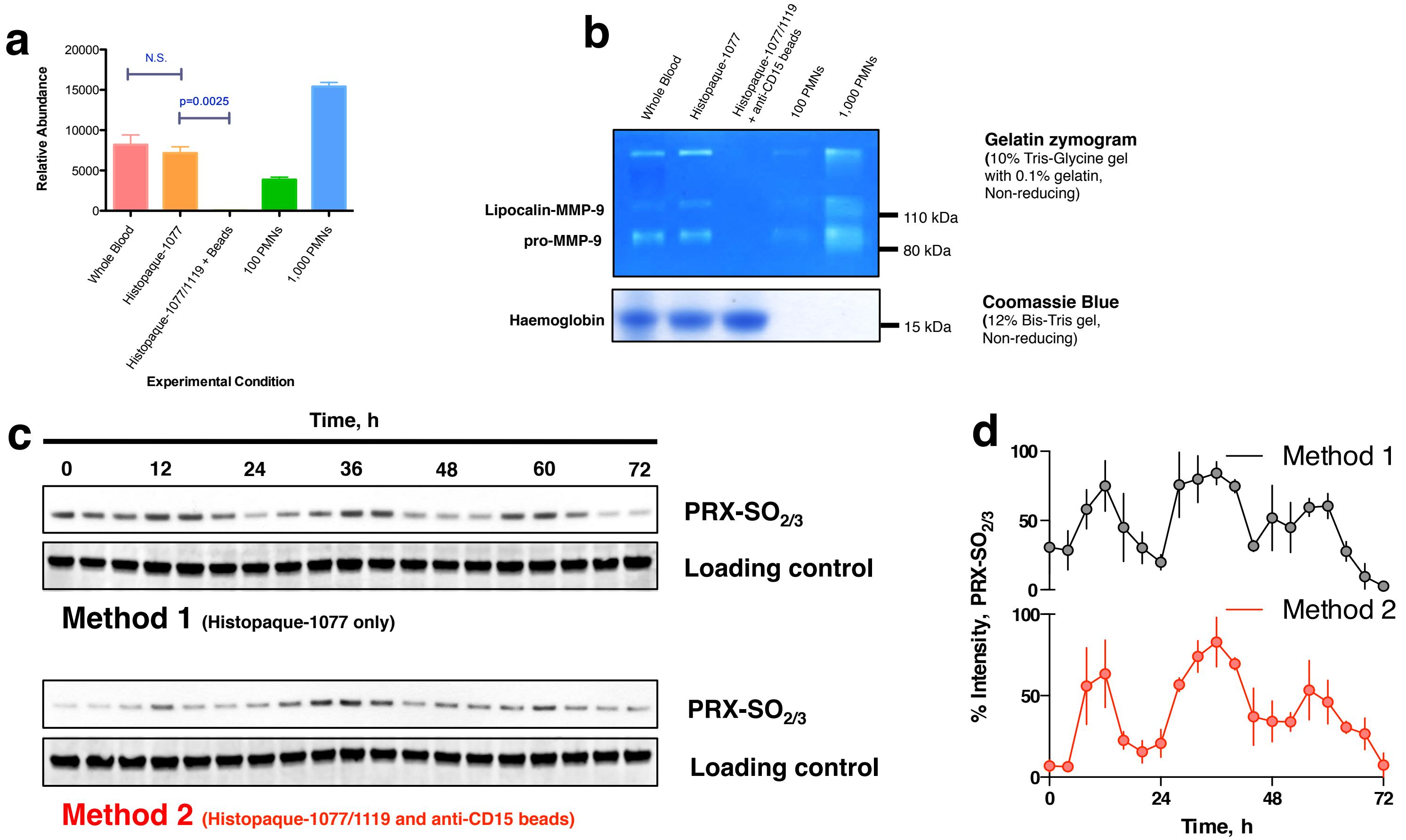

Supplementary Figure 3. Effect of granulocyte depletion on circadian rhythms in red blood cell preparations, related to Experimental Procedures.

(a) Grouped data comparing different methods of red blood cell purification by densitometry of gelatin zymograms (see b, n=3). Method 1 [Histopaque-1077 (Sigma-Aldrich) differential centrifugation only- and Method 2 [Histopaque-1077/1119 differential centrifugation followed by further granulocyte depletion using anti-CD15 magnetic Dynabeads (Invitrogen)]. Approximately 10^7 red cells were loaded per lane. Whole blood, 100 or 1,000 granulocytes were loaded for comparison. (b) Representative gelatin zymogram (top) showing granulocyte quantities from preparations in A, and coomassie blue stained gel to show hemoglobin loading (bottom). (c) Representative blots with anti-PRX-$SO_{2/3}$ antiserum comparing Method 1 and Method 2 in red cell preparations incubated under constant conditions (at 37°C in constant darkness, as previously) for the stated number of hours. (d) Quantification of blots from n=3 subjects by densitometry (Mean ± SEM). 2-way ANOVA: $p < 0.0001$ for time effect, N.S. for method of purification or interaction (time x method of purification).

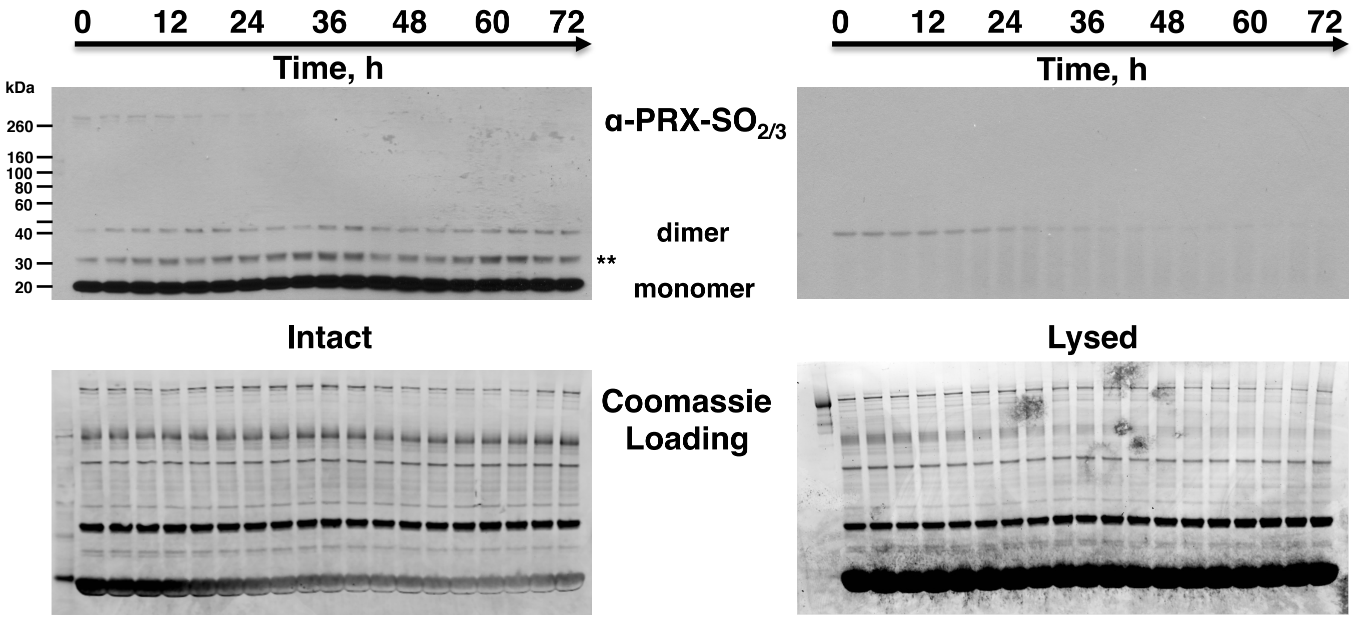

Supplementary Figure 4. Uncropped immunoblot scans from Figure 2.
Refer to supplementary figures 4 & 5 of reference[14] for further information; **non-specific.

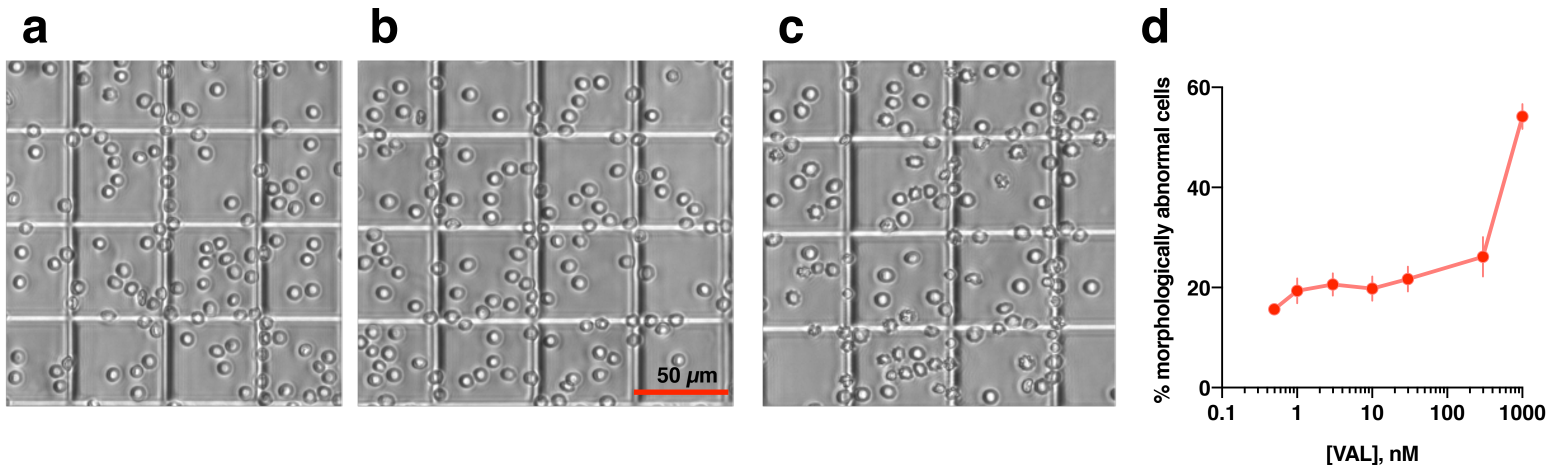

Supplementary Figure 5. Representative drug response curve and images of RBCs before and after a drug treatment, related to Experimental Procedures.
Control RBC morphology (a) after 96 hours *versus* 30 nM valinomycin (b) and 1 μM valinomycin (c). 1 μM valinomycin-treated cells exhibit a distinct change in morphology. (d) Data in the graph represent the average of isolated for 3 donors and incubated for 96 hours at the stated concentration of valinomycin. Damaged cells were those displaying morphology changes (e.g. crenation) or change in size. Cells from each donor and at each concentration were counted from 3 independent replicates. (Mean ± SEM). Unpaired t test: N.S for 30 nM and $p < 0.05$ at 1 μM.
